# Genetic Dissection of Quantitative Resistance to Common Rust (*Puccinia sorghi*) in Tropical Maize (*Zea mays* L.) by Combined Genome-Wide Association Study, Linkage Mapping, and Genomic Prediction

**DOI:** 10.3389/fpls.2021.692205

**Published:** 2021-07-02

**Authors:** Jiaojiao Ren, Zhimin Li, Penghao Wu, Ao Zhang, Yubo Liu, Guanghui Hu, Shiliang Cao, Jingtao Qu, Thanda Dhliwayo, Hongjian Zheng, Michael Olsen, Boddupalli M. Prasanna, Felix San Vicente, Xuecai Zhang

**Affiliations:** ^1^College of Agronomy, Xinjiang Agricultural University, Urumqi, China; ^2^International Maize and Wheat Improvement Center (CIMMYT), Texcoco, Mexico; ^3^College of Agronomy, Henan Agricultural University, Zhengzhou, China; ^4^College of Bioscience and Biotechnology, Shenyang Agricultural University, Shenyang, China; ^5^CIMMYT-China Specialty Maize Research Center, Crop Breeding and Cultivation Research Institute, Shanghai Academy of Agricultural Sciences, Shanghai, China; ^6^Maize Research Institute, Heilongjiang Academy of Agricultural Sciences, Harbin, China; ^7^Maize Research Institute, Sichuan Agricultural University, Chengdu, China; ^8^International Maize and Wheat Improvement Center (CIMMYT), Nairobi, Kenya

**Keywords:** maize, common rust, quantitative resistance, genome-wide association study, linkage mapping, genomic prediction

## Abstract

Common rust is one of the major foliar diseases in maize, leading to significant grain yield losses and poor grain quality. To dissect the genetic architecture of common rust resistance, a genome-wide association study (GWAS) panel and a bi-parental doubled haploid (DH) population, DH1, were used to perform GWAS and linkage mapping analyses. The GWAS results revealed six single-nucleotide polymorphisms (SNPs) significantly associated with quantitative resistance of common rust at a very stringent threshold of *P-*value 3.70 × 10^–6^ at bins 1.05, 1.10, 3.04, 3.05, 4.08, and 10.04. Linkage mapping identified five quantitative trait loci (QTL) at bins 1.03, 2.06, 4.08, 7.03, and 9.00. The phenotypic variation explained (PVE) value of each QTL ranged from 5.40 to 12.45%, accounting for the total PVE value of 40.67%. Joint GWAS and linkage mapping analyses identified a stable genomic region located at bin 4.08. Five significant SNPs were only identified by GWAS, and four QTL were only detected by linkage mapping. The significantly associated SNP of S10_95231291 detected in the GWAS analysis was first reported. The linkage mapping analysis detected two new QTL on chromosomes 7 and 10. The major QTL on chromosome 7 in the region between 144,567,253 and 149,717,562 bp had the largest PVE value of 12.45%. Four candidate genes of *GRMZM2G328500*, *GRMZM2G162250*, *GRMZM2G114893*, and *GRMZM2G138949* were identified, which played important roles in the response of stress resilience and the regulation of plant growth and development. Genomic prediction (GP) accuracies observed in the GWAS panel and DH1 population were 0.61 and 0.51, respectively. This study provided new insight into the genetic architecture of quantitative resistance of common rust. In tropical maize, common rust could be improved by pyramiding the new sources of quantitative resistance through marker-assisted selection (MAS) or genomic selection (GS), rather than the implementation of MAS for the single dominant race-specific resistance gene.

## Introduction

Common rust, caused by *Puccinia sorghi*, is one of the major foliar diseases in maize, which can cause up to 49% grain yield loss in susceptible varieties (Groth et al., 1983). The most sustainable strategy for controlling common rust is to develop and deploy resistant maize varieties, which requires the identification of the new source of resistance to common rust and the further understanding of the genetic basis and architecture of common rust resistance (Kibe et al., 2020).

In several recent studies, a broad genetic variation for common resistance was observed in tropical maize, and a few tropical maize inbred lines showing good resistance to common rust were identified (Rossi et al., 2020; Sserumaga et al., 2020). Among 50 tropical adapted maize breeding lines developed by International Maize and Wheat Improvement Center (CIMMYT), 12 lines with broad genetic diversity were identified as the potential donors of resistance alleles, and these lines are valuable breeding materials for the development and deployment of resistant hybrids to control common rust in tropical maize (Sserumaga et al., 2020). Furthermore, tropical maize germplasm is also an important source of resistance for improving common rust in temperate maize, and the six inbred lines developed by CIMMYT were identified as novel donors in Argentina for incorporating resistance to the local germplasm (Rossi et al., 2020). Those studies indicated the presence of genetic resistance to common rust in tropical maize germplasm. The donor lines identified in these studies are valuable donors for improving common rust resistance through breeding, which also are novel resistance sources for providing a better understanding of the genetic basis and architecture of common rust resistance.

Host-plant resistance, including both qualitative and quantitative resistances, had been identified as the most reliable and sustainable strategy for controlling common rust in maize (Zheng et al., 2018; Kibe et al., 2020). Previous efforts to exploit genetic resistance for common rust have largely been through dominant resistance (Rp) genes, and more than 26 Rp genes had been identified on maize chromosomes 3, 4, 6, and 10 (Hooker, 1985; Delaney et al., 1988). The Rp gene is qualitative and exhibits a high level of resistance to a specific *P. sorghi* race, and the resistance allele of Rp genes can be easily fixed into the breeding materials, but the resistance of Rp genes in some hybrids could break down due to the emerging *P. sorghi* race or multiple races caused infection happened in natural field condition (Zheng et al., 2018; Kibe et al., 2020). Quantitative resistance is due to partial or adult plant resistance, which is non-race-specific and often controlled by several genes to reduce the rate of fungal development on plant tissues (Olukolu et al., 2016). A few studies have been carried out on quantitative resistance to common rust mainly through linkage mapping (Lübberstedt et al., 1998; Kerns et al., 1999; Brown et al., 2001). Further studies are required to detect more sources of novel quantitative resistance alleles and exploit them to develop elite inbred lines or hybrids having stable and durable host-plant resistance to common rust.

Several linkage mapping analyses had been conducted in different genetic backgrounds to detect quantitative trait loci (QTL) associated with partial resistance to common rust (Lübberstedt et al., 1998; Kerns et al., 1999; Brown et al., 2001). These studies emphasized QTL detection in temperate maize germplasm, and QTL associated with partial resistance to common rust were distributed over all 10 chromosomes, without preference to chromosomes 3, 4, 6, and 10, which harbor qualitative Rp genes. Some QTL were overlapped in different studies and were consistent in different genetic backgrounds. These results suggest that major QTL associated with partial resistance from various elite backgrounds are possible to be pyramided for improving common rust resistance in temperate maize germplasm, and selection for multiple partial resistance alleles seems to be more promising than the marker-assisted selection (MAS) of the Rp genes.

Genome-wide association study (GWAS) is a useful tool for identifying molecular markers significantly associated with the target trait and exploring the underlying candidate genes (Yan et al., 2011; Wang et al., 2019). In a collection of 274 temperate maize inbred lines, the GWAS analysis was conducted to identify the SNPs significantly associated with common rust resistance; three loci significantly associated with common rust resistance were identified; and they were on chromosomes 2, 3, and 8. Candidate genes at these loci had predicted roles in cell wall modification and in regulating the accumulation of reactive oxygen species (Olukolu et al., 2016). The combined use of GWAS and linkage mapping can complement the strengths and weaknesses of each approach, and this approach has been successfully used in maize to dissect the genetic basis and architecture of complex traits (Li et al., 2016; Cao et al., 2017). In tropical maize germplasm, the combined use of GWAS and linkage mapping approach was applied to dissect the genetic basis of partial resistance to common rust recently (Zheng et al., 2018; Kibe et al., 2020). The results of these studies provide valuable information on understanding the genetic basis of common rust resistance; the common stable QTL regions identified by both GWAS and linkage mapping, and the major QTL identified by GWAS or linkage mapping individually need to be explored further for developing functional molecular markers for MAS.

Genomic selection (GS), also known as genomic prediction (GP), is an extension of MAS that uses genome-wide markers to predict the genomic estimated breeding values (GEBVs) of the unphenotyped lines for selection (Meuwissen et al., 2001; Crossa et al., 2014). GP can greatly accelerate the genetic gain per unit time and the cost in plant breeding programs for complex traits, and it has been reported in many studies (Gowda et al., 2015; Zhang et al., 2015; Beyene et al., 2019; Wang et al., 2020a). To our knowledge, only one study has been reported evaluating the potential of GS and GP for improving common rust resistance in maize, where the GP accuracies ranged from 0.19 to 0.51 in different populations (Kibe et al., 2020).

In this study, a GWAS panel and a bi-parental DH population were used to perform GWAS, linkage mapping, and GP analyses, where both populations were phenotyped in multi-environment trials to evaluate their responses to common rust and genotyped with genotyping-by-sequencing (GBS) single-nucleotide polymorphisms (SNPs). The main objectives of this study were to: (1) detect the significantly associated SNPs, major QTL, and putative candidate genes conferring common rust resistance in tropical maize by the combined use of GWAS and linkage mapping; (2) explore the potential of GS and GP for improving common rust resistance; and (3) estimate the GP accuracies under different factors affecting the accuracy estimation.

## Materials and Methods

### Plant Materials

A GWAS panel of 282 genetically diverse inbred lines was used for the GWAS and GP analyses in this study (Supplementary Table 1). The GWAS panel, Drought Tolerant Maize for Africa (DTMA), was collected by the Global Maize Program of CIMMYT. Based on the geographic information and environmental adaptation, the DTMA panel can be classified into nine subsets: (1) breeding lines from the lowland tropical maize breeding program in Mexico, (2) breeding lines from the highland tropical maize breeding program in Mexico, (3) breeding lines from the subtropical maize breeding program in Mexico; (4) inbred lines from the maize physiology breeding program in Mexico, (5) inbred lines from the maize entomology breeding program in Mexico, (6) breeding lines from the lowland tropical maize breeding program in Colombia, (7) breeding lines from the mid-altitude maize breeding program in Zimbabwe, (8) breeding lines from the highland tropical maize breeding program in Ethiopia, and (9) breeding lines from the maize breeding program of International Institute of Tropical Agriculture in Nigeria (Cairns et al., 2013; Yuan et al., 2019). A bi-parental DH population, DH1, was used for the linkage mapping and GP analyses. This DH population consisted of 189 DH lines, which were derived from the F_1_ cross formed with two elite inbred lines of CML495 and La Posta Sequia C7 F64-2-6-2-2-B-B-B, CML495 shows good resistance to common rust, and La Posta Sequia C7 F64-2-6-2-2-B-B-B is susceptible to common rust.

### Experimental Design

Both populations were evaluated for response to common rust under consistently high natural disease pressure at several locations in Mexico. The DTMA panel was evaluated at Agua Fria in the state of Puebla (110 masl; mega-environment: lowland tropical) in 2008, 2009, 2010, and 2012. Two tropical maize inbred lines (B.T.Z.T.R.L.BA90 12-1-1P-1P-1-1-1-1P-1-B/BTZTVCPR92A 27-7P-1-1P-1P-4P-B-B)-B-60TL-1-1-B-B-B-B and CML139 were used in all the trials as the resistant and susceptible checks, respectively. The population of DH1 was evaluated in two locations in 2013 at El Batan in the state of Mexico (2,249 masl; mega-environment: highland tropical) and Santa Catarina in the state of Nuevo Leon (680 masl; mega-environment: subtropical), respectively. For the DH1 population, the parental lines were used as the resistant and susceptible checks. A randomized complete block design with three replications was used for all trials. Each plot consisted of 11 plants in a 2 m row with a width of 0.75 m.

### Disease Evaluation

Plants were visually evaluated for common rust three times at 7-day intervals, beginning 2 weeks after flowering. Disease severity was evaluated on a 1–5 scale based on the percentage of leaf area covered by lesions. A rating scale of 1 corresponds to high resistance covering 0–10% of the leaf surface, 2 corresponds to weak to moderate infection covering 10–25% of the leaf surface, 3 corresponds to moderate infection covering 25–50% of the leaf surface, 4 corresponds to moderate-to-severe infection covering 50–75% of the leaf surface, and 5 corresponds to severe infection covering > 75% of the leaf surface. For each plot, the final highest score was used for further analysis. In both the DTMA panel and the DH1 population, the resistant and susceptible checks were used as controls to check for adequate levels of disease infection.

### Phenotypic Data Analysis

The multi-environment trial analysis was conducted using META-R Version 6.04 (Alvarado et al., 2020). A mixed linear model was used to calculate the best linear unbiased predictors (BLUPs), variance components, and broad-sense heritability. The model used for data analysis was as follows:

(1) $${\text{Y}}_{i\,j\,k}=\mu +{\text{G}}_{k}+{\text{E}}_{i}+{\text{R}}_{j\,\left(i\right)}+{\text{EG}}_{i\,k}+\varepsilon_{i\,j\,k}$$

where *Y*_*ijk*_ is the observation of the *k*th genotype in the *i*th environment in the *j*th replicate, μ is the overall mean, *G*_*k*_ is the effect of the *k*th genotype, *E*_*i*_ is the effect of the *i*th environment, *R*_*j(i)*_ is the effect of the *j*th replication nested on the *i*th environment, EG*_*ik*_* is the effect of the interaction between the *i*th environment and *k*th genotype, and ε*_*ijk*_* is the effect of experimental error. BLUPs across all environments were used for GWAS, linkage mapping, and GP analyses. Broad-sense heritability across all environments was calculated as follows:

(2) h2=σg2σg2+σge2i+σe2ij

where $\sigma_{g}^{2}$ is the genotypic variance, $\sigma_{g\,e}^{2}$ is the genotype × environment interaction variance, $\sigma_{e}^{2}$ is the error variance, *i* is the number of environments, and *j* is the number of replications in each environment. All of the factors were set as random effects when calculating heritability.

### Genotyping and Genotypic Data Analysis

Young leaves of all the inbred lines and the parental lines were sampled for both populations. DNA extraction was performed using a CTAB method (CIMMYT, 2005). Genotypic data was generated using the GBS method at the Cornell University Biotechnology Resource Center (Ithaca, NY, United States). DNA sequencing was performed on Illumina HiSeq2000. TASSEL GBS Pipeline was used for SNP calling to align reads to maize B73 reference genome v2 (ZmB73_RefGen_v2). Imputation was carried out with the FILLIN method in TASSEL V5.0 (Bradbury et al., 2007; Swarts et al., 2014). The imputed GBS dataset was used for the GWAS and GP analyses, while the unimputed GBS dataset was used for the linkage mapping analysis (Wang et al., 2020b). A total of 955,690 SNPs were obtained for each inbred line, and 570 of them could not be mapped to any of the 10 maize chromosomes. The number of SNPs on each chromosome ranged from 148,752 on chromosome 1 to 67,126 on chromosome 10. SNPs with the missing rate (MR) of >20%, the heterozygosity rate of >5%, and the minor allele frequency (MAF) of <0.05 were excluded using the filter function in TASSEL V5.0.

### Analyses of Linkage Disequilibrium, Population Structure, and GWAS

After filtering, 187,409 SNPs were obtained for GWAS in the DTMA panel. The linkage disequilibrium (LD) analysis was carried out using TASSEL V5.0 with a sliding window size of 50 SNPs. A squared Pearson correlation coefficient (*r*^2^) between the vectors of SNP alleles was used to assess the level of LD decay across each chromosome, and *r*^2^ = 0.1 was used as a cutoff. Population structure was conducted using the STRUCTURE V2.3.4 software (Hubisz et al., 2009) to estimate the number of subgroups in the DTMA panel, where one SNP per LD block was selected for the following analysis (Duggal et al., 2008). The parameters were set as follows: length of burn-in period = 30,000, number of MCMC reps after burn-in = 30,000, ancestry model = use admixture model, allele frequency model = allele frequency correlated, number of populations (*K*) = 1–10, and number of iterations = 10. STRUCTURE HARVESTER (Earl and vonHoldt, 2012) was used to visualize STRUCTURE V2.3.4 output, and delta *K* (Δ*K*) value was used to determine the *K* value of the number of subgroups.

Analysis of GWAS was conducted in the DTMA panel using the Fixed and random model Circulating Probability Unification (FarmCPU) method (Liu et al., 2016) in Genome Association and Prediction Integrated Tool-R (GAPIT) package (Lipka et al., 2012). The kinship matrix and the first three PCs were estimated by GAPIT to assess the population structure and control the false marker-trait association. The *P-*value of each SNP was calculated, and the threshold of *P-*value was determined at 3.70 × 10^–6^ by a false discovery rate correction method. The 100 bp source sequences of each significant SNP were used to do BLAST against the ZmB73_RefGen_v2 genome sequence in MaizeGDB (Portwood et al., 2019). Within the local LD block of significant SNPs, the annotated genes that are likely involved in disease resistance were identified as the putative candidate genes.

### Linkage Map Construction and Linkage Mapping Analysis

A similarity/linkage (SL) method was used for bin map construction with high-quality unimputed SNPs in the DH1 population, and the details were previously described by Cao et al. (2017). In brief, 437 bins were constructed by 31,194 SNPs. Each bin was regarded as a genetic marker to construct the linkage map. Linkage map construction was conducted by MAP function in QTL IciMapping V4.2 software (Meng et al., 2015). The whole length of the linkage map of DH1 was 988.56 cM with an average marker (bin) density of 2.26 cM. An inclusive composite interval mapping (ICIM) approach was conducted for the linkage mapping analysis using the “BIP” function and the “ADD” mapping method in QTL IciMapping V4.2. A logarithm of the odds (LOD) score of 3.0 was used to declare the putative QTL. The additive effect and phenotypic variation explained (PVE) of each QTL were estimated.

### Genomic Prediction Analysis

Genomic prediction analysis was conducted using the ridge regression best linear unbiased prediction (RRBLUP) model with the rrBLUP package (Endelman, 2011) within the DTMA panel and the DH1 population. In the imputed GBS dataset, TASSEL version 5.0 was used to filter the SNPs with a MAF > 0.05, a MR < 20%, and a heterozygosity rate < 5%. After filtering, 187,409 and 53,996 SNPs were used for GP in the DTMA panel and the DH1 population, respectively. In the DH1 population, 437 bins were also used for the GP analysis to estimate the prediction accuracy and compared it with the prediction accuracy estimated using all the 53,996 SNPs. To estimate the effect of marker density on GP accuracy, the number of SNPs varied from 100 to 50,000 (i.e., 10, 50, 100, 300, 500, 1,000, 3,000, 5,000, 10,000, and 50,000) were used to estimate the prediction accuracy in the DTMA panel and the DH1 population. In each marker density, SNPs were randomly selected 100 times. A fivefold cross-validation scheme repeated 100 times was used to estimate the prediction accuracy, where the prediction accuracy was defined as the average value of the correlations between the GEBVs and the observed breeding values. Training population size (TPS), ranged from 10 to 90% of the total population size, was selected to assess the effect of TPS on prediction accuracy in each of the two populations. The training set was randomly sampled to predict, and the remaining lines were used as the prediction set. The GP analysis was repeated 100 times in each population with different TPS.

## Results

### Phenotypic Variations

The descriptive statistics for the response to common rust in the DTMA panel and the DH1 population are presented in Table 1 and Supplementary Figure 1A. The results indicated that there were abundant phenotypic variations within each population. In the DTMA panel, the disease scores ranged from 1.26 to 4.13, with a mean of 2.32. In the DH1 population, the disease scores ranged from 1.73 to 3.10, with a mean of 2.25. The most resistant (top 10%) and most susceptible lines (bottom 10%) for common rust in the DTMA panel and the DH1 populations are shown in Supplementary Tables 2, 3, respectively. The mixed model analysis result revealed that the genotypic variance was statistically highly significant at *P* < 0.01 in both populations, as well as the variance of genotype-by-environment interaction. The estimated broad-sense heritabilities in the DTMA panel and the DH1 population were 0.80 and 0.57, respectively.

**TABLE 1:** Descriptive statistics, variance components, and broad-sense heritability (*H*^2^) response to common rust in the Drought Tolerant Maize for Africa (DTMA) panel and the bi-parental doubled haploid (DH1) population.

Population	No. of lines	Mean	Min.	Max.	Median	SD^*a*^	Variance components^*b*^			*h^2*c*^*
	
							$\sigma_{g}^{2}$	$\sigma_{g\,e}^{2}$	$\sigma_{e}^{2}$	
DTMA	282	2.32	1.26	4.13	2.30	0.52	0.33**	0.25**	0.24	0.80
DH1	189	2.25	1.73	3.10	2.20	0.23	0.10**	0.08**	0.20	0.57

*^*a*^SD, standard deviation.*

*^*b*^$\sigma_{g}^{2}$, genotypic variance.*

*$\sigma_{g\,e}^{2}$, genotype × environment interaction variance.*

$\sigma_{e}^{2}$, error variance.

**Significant at *P* < 0.01.

*^*c*^*h*^2^, broad-sense heritability.*

### Basic Information of SNPs Before and After Filtering

The basic information about GBS data before and after filtering is shown in Supplementary Table 4. The number of SNPs after filtering decreased from 955,690 to 187,409 in the imputed dataset of the DTMA panel and from 955,690 to 31,194 in the unimputed dataset of the DH1 population. The MR after filtering decreased from 15.79 to 7.33% in the imputed dataset of the DTMA panel and from 42.53 to 9.73% in the unimputed dataset of the DH1 population. The heterozygosity rate increased in both populations after filtering, and the heterozygosity rates after filtering in the DTMA panel and the DH1 population were 2.83 and 3.17%, respectively. The average MAF after filtering increased from 0.09 to 0.18 in the DTMA panel and from 0.04 to 0.42 in the DH1 population.

### Results of LD Decay Distance and Population Structure in the DTMA Panel

**FIGURE 1:**
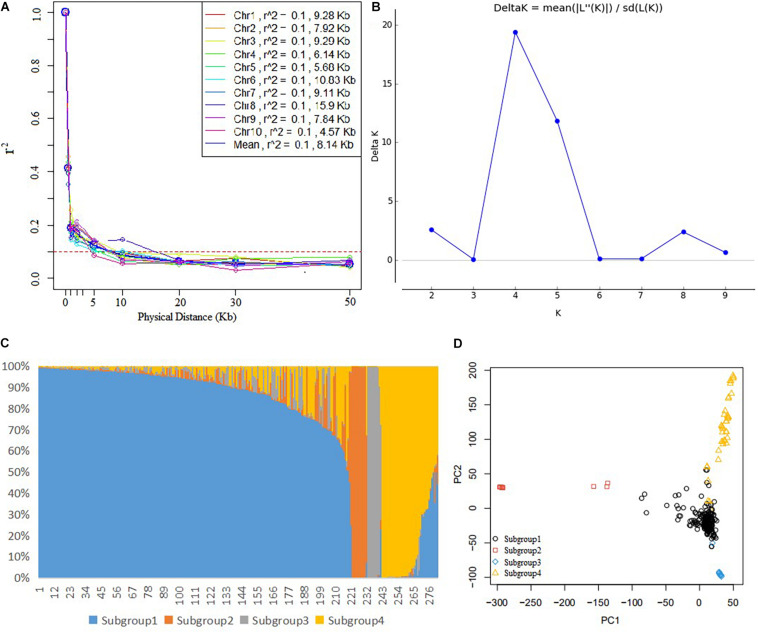
Analysis of genetic diversity in the genome-wide association study (GWAS) panel. **(A)** Linkage disequilibrium decay across all 10 maize chromosomes, **(B)** the plot of delta *K*, **(C)** the estimated probability membership for each inbred line at *K* = 4, and **(D)** the principal component analysis plot showing four subgroups corresponding to the four subgroups by the STRUCTURE analysis.

In the DTMA panel, the average LD decay distance across all the 10 chromosomes was 8.14 kb at an *r*^2^ value of 0.1 (Figure 1A), and it ranged from 4.57 kb in chromosome 10–15.9 kb in chromosome 8. The population structure analysis showed that the delta *K* value reached a peak when the *K* value was 4, indicating that the DTMA panel can be divided into four subgroups (Figures 1B,C). The number of lines in subgroups 1, 2, 3, and 4 was 219, 13, 10, and 40, respectively. The different responses to common rust in the four subgroups are shown in Supplementary Figure 1B. The principal component analysis also revealed four subgroups, corresponding to the four subgroups identified by STRUCTURE analysis (Figure 1D).

### Significantly Associated SNPs and Candidate Genes Revealed by GWAS

**FIGURE 2:**
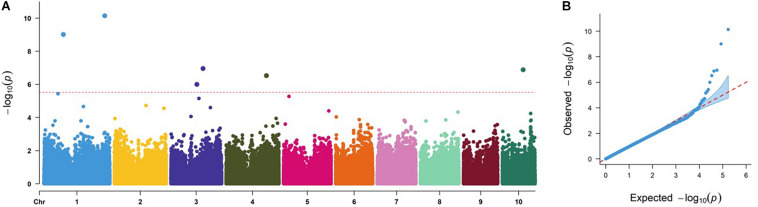
Genome-wide association study Manhattan and quantile–quantile (q–q) plots for common rust resistance in the Drought Tolerant Maize for Africa (DTMA) panel. **(A)** Manhattan plot, the dashed line corresponds to the threshold level defined at *P* = 3.70 × 10^–6^ by a false discovery rate correction method; **(B)** q–q plot.

The GWAS results of the DTMA panel are presented in Table 2 and Figure 2. At a very stringent threshold of *P-*value of 3.70 × 10^–6^, a total of six SNPs at bins of 1.05, 1.10, 3.04, 3.05, 4.08, and 10.04 were identified to be significantly associated with common rust resistance in maize. The quantile–quantile (q–q) plot implied that the population structure and family relatedness were well controlled in the GWAS using the FarmCPU method (Figure 2B).

**TABLE 2:** Significantly associated single-nucleotide polymorphisms (SNPs) and candidate genes revealed by the genome-wide association study analysis.

SNP^*a*^	*P-*value	Allele^*b*^	MAF^*c*^	SNP effect^*d*^	Putative candidate gene	Annotation of candidate genes
S1_89238026	9.81 × 10^–10^	A/G	0.32	0.13	*GRMZM2G114893*	Zinc finger (C2H2 type) family protein
S1_278132829	7.25 × 10^–11^	A/T	0.25	0.13	*GRMZM2G328500*	UDP-glucose 6-dehydrogenase
S3_118933375	1.00 × 10^–6^	C/T	0.10	−0.17	*GRMZM2G144004*	Unknown
S3_147594533	1.11 × 10^–7^	A/T	0.11	0.15	*GRMZM2G162250*	*Zea mays ARGOS6*
S4_183913302	2.98 × 10^–7^	G/C	0.17	0.13	*GRMZM2G138949*	BTB/POZ domain-containing protein
S10_95231291	1.32 × 10^–7^	C/A	0.10	−0.16	*GRMZM2G131536*	Unknown

*^*a*^SNP name, chromosome_position, for example, S1_89238026 represents that the SNP is on chromosome 1, and the physical position is 89238026 bp.*

*^*b*^Letters to the left and right of the “/” refer to major allele and minor allele, respectively.*

*^*c*^MAF, minor allele frequency.*

*^*d*^Positive values indicate that the major allele is a resistance allele, and the negative values indicate that the minor allele is a resistance allele.*

Among all the six SNPs, the two most significantly associated SNPs were identified on chromosome 1. The most significantly associated SNP of S1_278132829 was located at the bin of 1.10, it had the lowest *P*-value of 7.25 × 10^–11^, and the MAF of this SNP was 0.25, with an additive effect of 0.13. The candidate gene of *GRMZM2G328500* (278,126,093–278,132,841 bp), encoding a UDP-glucose 6-dehydrogenase, contains the most significantly associated SNP of S1_278132829. The second most significantly associated SNP of S1_89238026 was located at the bin of 1.05, it had the second-lowest *P*-value of 9.81 × 10^–10^, and the MAF of this SNP was 0.32, with an additive effect of 0.13. It neighbored with the candidate gene of *GRMZM2G114893* (89,236,681–89,237,918 bp), which encodes a zinc finger (C_2_H_2_ type) family protein.

On chromosome 3, two significantly associated SNPs were identified, i.e., S3_118933375 located at the bin of 3.04 and S3_147594533 located at the bin of 3.05. The SNP of S3_118933375 had a MAF of 0.10, with an additive effect of −0.17, and it was 587 bp away from the candidate gene of *GRMZM2G144004* (118,931,829–118,932,788 bp), encoding a putative uncharacterized protein. The SNP of S3_147594533 had a MAF of 0.11, with an additive effect of 0.15, and it was located at the candidate gene of *GRMZM2G162250* (147,591,043–147,598,482 bp), which encodes a *Zea mays* ARGOS6 (auxin-regulated gene involved in organ size) protein.

On chromosome 4, the significantly associated SNP of S4_183913302 was located at the bin of 4.08, it had a MAF of 0.17, with an additive effect of 0.13, and this SNP was close to the candidate gene of *GRMZM2G138949* (183,909,192–183,910,514 bp), encoding a BTB/POZ domain-containing protein. On chromosome 10, the significantly associated SNP of S10_95231291 was located at the bin of 10.04, it had a MAF of 0.10, with an additive effect of −0.16, and this SNP was closely linked with the candidate gene of *GRMZM2G131536* (95,230,282–95,231,024 bp).

### Quantitative Trait Loci Detected From Linkage Mapping Analysis

The linkage mapping results of the DH1 population are presented in Table 3. In total, five QTL located at bins 1.03, 2.06, 4.08, 7.03, and 9.00 were detected at the threshold of a LOD score of 3.0. The PVE value of the individual QTL ranged from 5.40 to 12.45%, and the total PVE value for all the five QTL was 40.67%. The QTL on chromosome 7 had the highest LOD score of 7.82 and the largest PVE value of 12.45%, indicating that it is a major QTL conferring the common rust resistance in maize. The common rust resistance alleles were derived from the resistant inbred line CML495 except for the two QTL located on chromosomes 1 and 9.

**TABLE 3:** Quantitative trait loci detected from the linkage mapping analysis in the doubled haploid (DH1) population.

Chromosome	Position (cM)	Bin	Left marker^*a*^	Right marker	LOD^*b*^	PVE(%)^*c*^	Additive effect
1	28	1.03	S1_31252133	S1_34315390	6.77	10.34	−0.08
2	47	2.06	S2_183941772	S2_188133361	3.49	5.69	0.06
4	74	4.08	S4_184936775	S4_186039203	4.62	6.79	0.06
7	67	7.03	S7_144567253	S7_149717562	7.82	12.45	0.09
9	0	9.00	S9_1260192	S9_2825523	3.70	5.40	−0.06

*^*a*^Marker name, chromosome_position.*

*^*b*^LOD, logarithm of the odds.*

*^*c*^PVE, phenotypic variation explained.*

The significantly associated SNP of S4_183913302 identified by GWAS was closely linked with the QTL detected in DH1 on chromosome 4, it was flanked by the markers S4_184936775 and S4_186039203, and this QTL had a LOD score of 4.62 and a PVE value of 6.79%. However, the most significantly associated SNP of S1_278132829 identified by GWAS was not validated by the linkage mapping analysis. The major QTL on chromosome 7 detected from linkage mapping analysis was also not validated by the GWAS result.

### Prediction Accuracies Estimated With the Different Marker Datasets, Marker Density, and Training Population Size

**FIGURE 3:**
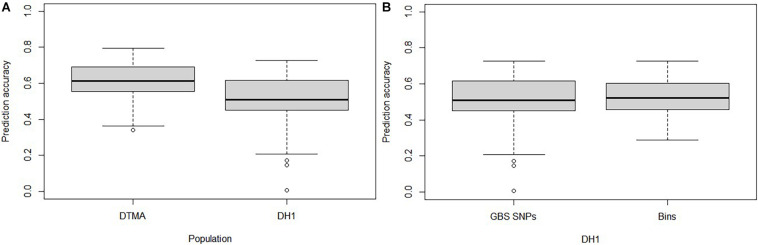
Genomic prediction accuracy of common rust resistance in the DTMA panel and DH1 population. **(A)** In the DTMA panel and DH1 population estimated with genotyping-by-sequencing (GBS) single-nucleotide polymorphisms (SNPs); **(B)** in the DH1 panel estimated with GBS SNPs and bins.

**FIGURE 4:**
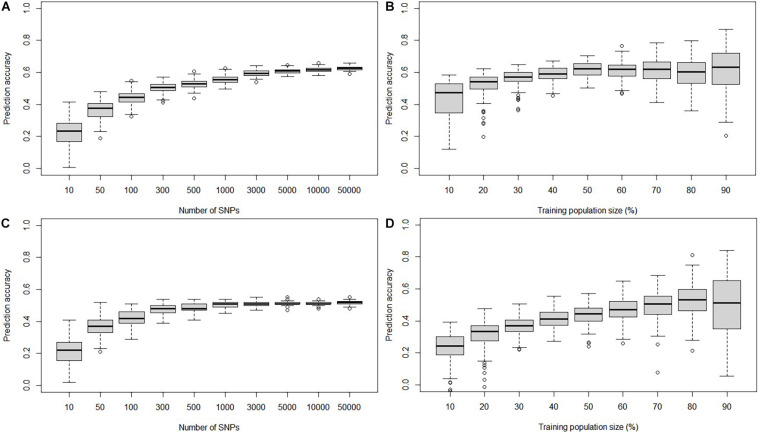
Genomic prediction accuracy of common rust resistance in the DTMA panel and DH1 population, when the number of SNPs varied from 100 to 50,000 and training population size (TPS) ranged from 10 to 90% of the total population size. **(A)** In the DTMA panel estimated with different marker density, **(B)** in the DTMA panel estimated with different TPS, **(C)** in the DH1 population estimated with different marker density, and **(D)** in the DH1 population estimated with different TPS.

The GP accuracies estimated based on GBS SNPs were 0.61 and 0.51 in the DTMA panel and the DH1 population, respectively (Figure 3A). The GP accuracy based on bin markers was 0.53 in DH1 (Figure 3B). No significant difference in prediction accuracy was observed between GBS SNPs and bin markers. The effect of marker density and TPS on the GP accuracy is shown in Figure 4. In both the DTMA panel and the DH1 population, the prediction accuracy increased as the number of markers increased. In the DTMA panel, the prediction accuracy increased rapidly when the number of markers increased from 10 to 5,000, and then, the prediction accuracy increased slightly when the number of markers kept increasing. In the DH1 population, a sharp increase in the prediction accuracy was observed before reaching a plateau at about 300 markers, indicating that 300 SNPs were sufficient to achieve good accuracy of common rust resistance in the DH1 population. Prediction accuracy increased as the TPS increased in both populations. In the DTMA panel, the prediction accuracy increased rapidly when the TPS increased from 10 to 50%, and then, a little improvement in the prediction accuracy was observed when the TPS kept increasing. When 50% of the total genotypes were used as the training set, a relatively high prediction accuracy coupled with the smaller standard error was observed. A similar trend was observed in the DH1 population.

## Discussion

Common rust is a major disease of maize, causing 34% of the maize area to suffer economic losses (Zheng et al., 2018). Developing maize varieties with host plant resistance is the most sustainable strategy for the control of common rust, which requires further understanding of the genetic basis and architecture of common rust resistance. Previous efforts to exploit genetic resistance for common rust have largely been through Rp genes, but the resistance of Rp genes could break down easily. Quantitative disease resistance controlled by several genes has proven to be highly durable, making it a better choice for long-term common rust resistance breeding. In this study, GWAS and linkage mapping analyses were applied to dissect the genetic base of quantitative resistance of common rust in maize. GWAS revealed six SNPs significantly associated with quantitative resistance of common rust at a very stringent threshold of *P-*value of 3.70 × 10^–6^. Linkage mapping identified five QTL accounting for the total PVE value of 40.67%. These results provided new insight into the quantitative resistance of common rust, which implied that major QTL associated with quantitative resistance from various elite backgrounds are possible to be pyramided for improving common rust resistance, and the selection for multiple partial resistance alleles seems to be more promising than the MAS of the Rp genes in tropical maize germplasm.

In the GWAS, six SNPs distributed in bins 1.05, 1.10, 3.04, 3.05, 4.08, and 10.04 were associated with common rust resistance. Except for SNP of S10_95231291, all the SNPs were reported in previous GWAS and linkage mapping studies (Lübberstedt et al., 1998; Brown et al., 2001; Zheng et al., 2018; Kibe et al., 2020). The most and the second most significantly associated SNPs S1_278132829 and S1_89238026 detected in this study were also detected by linkage mapping in European flint germplasm (Lübberstedt et al., 1998). SNP S3_118933375 was in the same region of qCR3-113, a QTL for common rust (Kibe et al., 2020), and it was also close to SNP PZE-103072633 (115,864,889) (Zheng et al., 2018). Both qCR3-113 and PZE-103072633 were detected in tropical maize germplasm. SNPs S3_147594533 and S4_183913302 were mapped to the QTL intervals associated with common rust in sweet corn (Brown et al., 2001). QTL detected for a target trait are usually different due to the use of different genetic backgrounds and environments (Ren et al., 2020). Those common loci detected in different studies were stable QTL for common rust. SNP S10_95231291 was first reported, it had an additive effect of −0.16, and it was closely linked with the candidate gene of GRMZM2G131536. However, the function of the candidate gene of GRMZM2G131536 is still unknown.

In DH1, linkage mapping revealed five QTL distributed in bins 1.03, 2.06, 4.08, 7.03, and 9.00, respectively. Three of the five QTL were reported previously (Lübberstedt et al., 1998; Brown et al., 2001). The loci in bins 1.03 and 2.06 coincided with QTL identified by Lübberstedt et al. (1998). The locus in bin 4.08 was detected by both Lübberstedt et al. (1998) and Brown et al. (2001). The major QTL located on chromosome 7 was reported in this study for the first time, and it had the highest LOD score of 7.82 and the largest PVE value of 12.45%. It is a new source of resistance for common rust, which deserves further investigation.

Joint GWAS and linkage mapping can complement the advantages and disadvantages of each method (Li et al., 2016; Cao et al., 2017). In this study, GWAS and linkage mapping were implemented stepwise to detect loci associated with quantitative resistance of common rust. The genomic region located at bin 4.08 was detected by both GWAS and linkage mapping. SNP S4_183913302 was consistent with the locus identified between markers S4_184936775 and S4_186039203 in DH1. This locus was also reported by Lübberstedt et al. (1998) and Brown et al. (2001). The major QTL located on chromosome 7 identified by linkage mapping in DH1 was not detected through GWAS in the DTMA panel. This may be due to the very low frequency of one of the alleles of the relevant locus in the GWAS panel or the population structure related to the polymorphism at this locus (Famoso et al., 2011; Cadic et al., 2013). The most significantly associated SNP of S1_278132829 identified by GWAS was also not validated by the linkage mapping analysis. It may be because there is no genetic variation at this locus in the DH1 population. The major QTL identified by GWAS or linkage mapping individually, and the common stable QTL region identified by both methods need to be explored further for developing functional molecular markers for MAS.

The candidate gene analysis can lead to a better understanding of the genetic basis of common rust resistance. According to the results of GWAS, six candidate genes were identified in this study, and the function of four candidate genes was annotated. These candidate genes were previously reported to play important roles in the response of stress resilience and the regulation of plant growth and development. *GRMZM2G328500* encodes a UDP-glucose 6-dehydrogenase, which is involved in the nucleotide-sugar interconversion process (Kost et al., 2020). *GRMZM2G162250* encodes a *Zea mays* ARGOS6 protein controlling plant growth, organ size, and grain yield. *GRMZM2G114893* encodes a zinc finger (C_2_H_2_ type) family protein, which is mainly involved in the regulation of plant growth, development, and tolerance to biotic and abiotic stresses (Kim et al., 2009; Xiao et al., 2009). *GRMZM2G138949* identified in bin 4.08 encodes a BTB/POZ domain-containing protein, which participates in a series of physiological and biochemical reactions and also plays an important role in resistance to plant disease (Cao et al., 1997; Silva et al., 2015). These results encourage fine-mapping and cloning of the candidate genes for controlling common rust in maize.

Genomic prediction and GS have been successfully used in several crops to accelerate genetic gain in breeding programs for improving complex traits, including resistance to major maize diseases (Gowda et al., 2015; Liu et al., 2021). A study on the potential of GS and GP to improve the common rust resistance in maize has been reported by Kibe et al. (2020), where the GP accuracies within populations ranged from 0.19 to 0.51, and the GP accuracies estimated from a larger population by combined several individual populations were higher than those estimated from the individual population with a smaller size. For implementing GP and GS to improve common rust resistance in tropical maize, an independent but related training set is encouraged to be built to predict the related populations not been phenotyped. These results were confirmed by this study. The GP accuracies observed in the DTMA panel and the DH1 population were 0.61 and 0.51, respectively. It indicates that common rust resistance in tropical maize could be improved by implementing GP and GS. Moreover, the factors affecting GP accuracy were also assessed in this study. Ten levels of marker density were used to assess the effect of marker density on prediction accuracy in the two populations. The results showed that the increase in marker density leads to an increase in prediction accuracy. The prediction accuracy reached a plateau when the marker density was 5,000 in the DTMA panel and 300 in the DH1 panel, which indicated that more makers are required to achieve good GP accuracy in populations with higher genetic diversity. A similar phenomenon was found for several traits in maize (Zhang et al., 2017; Guo et al., 2020; Liu et al., 2021). There was no significant difference between the prediction accuracy estimated based on the GBS SNPs and the bins in the DH1 population, which validated the high quality and accuracy of bins constructed in the bi-parental population. To assess the effect of TPS on prediction accuracy, nine levels of TPS were selected. As a result, increasing TPS leads to an increase in prediction accuracy. When 50% of the total genotypes were used as the training set, a relatively high prediction accuracy can be achieved. These results provide valuable information for improving common rust resistance in tropical maize by implementing GP and GS.

## Data Availability Statement

The original contributions presented in the study are publicly available. These data can be found at the CIMMYT Research Data & Software Repository Network: https://hdl.handle.net/11529/10548575.

## Author Contributions

BP, MO, TD, FS, and XZ conceived and designed the experiments. TD, FS, and XZ coordinated the phenotyping. XZ, BP, and MO coordinated the genotyping. JR, ZL, PW, AZ, YL, GH, SC, JQ, and HZ analyzed the data. JR, ZL, FS, and XZ drafted the manuscript. JR, ZL, MO, BP, FS, and XZ interpreted the results. All authors contributed to the article and approved the submitted version.

## Conflict of Interest

The authors declare that the research was conducted in the absence of any commercial or financial relationships that could be construed as a potential conflict of interest.
