# Supplementary material for: Genetic Dissection of Quantitative Resistance to Common Rust (Puccinia sorghi) in Tropical Maize (Zea mays L.) by Combined Genome-Wide Association Study, Linkage Mapping, and Genomic Prediction
Source: Front Plant Sci. 2021 Jul 2;12:692205. doi: 10.3389/fpls.2021.692205 (PMC8284423; doi:10.3389/fpls.2021.692205)
Supplement: Supplementary file 1 [file Data_Sheet_1.docx]

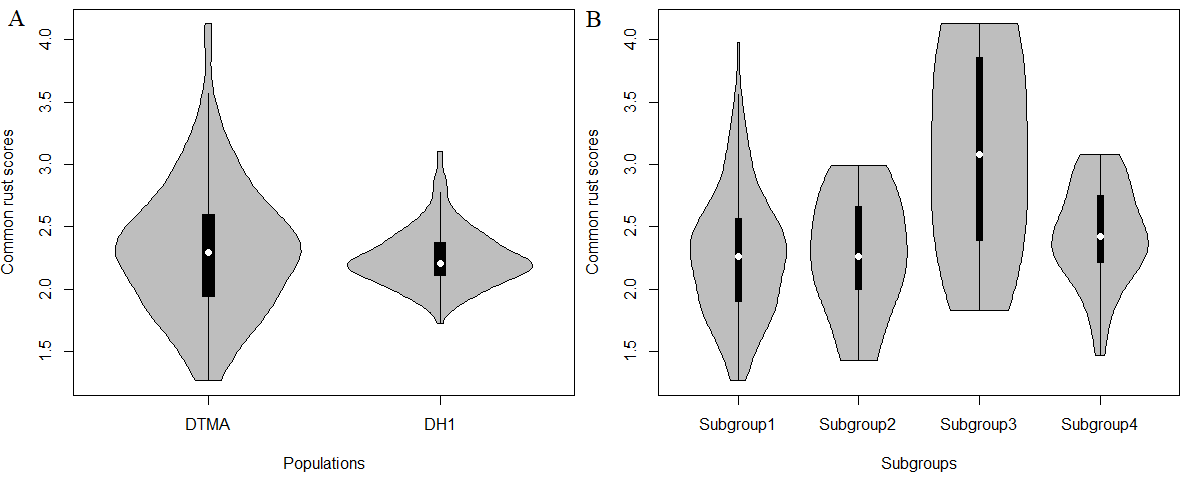


Figure S1. Violin plots of common rust scores in the (A) Drought Tolerant Maize for Africa (DTMA) panel and DH1 population and (B) subgroups of the DTMA panel.
