# Supplementary material for: Genetic Dissection of Quantitative Resistance to Common Rust (Puccinia sorghi) in Tropical Maize (Zea mays L.) by Combined Genome-Wide Association Study, Linkage Mapping, and Genomic Prediction
Source: Front Plant Sci. 2021 Jul 2;12:692205. doi: 10.3389/fpls.2021.692205 (PMC8284423; doi:10.3389/fpls.2021.692205)
Supplement: Supplementary file 3 [file Table_2.DOCX]

Table S2. The most resistant (top 10% ) and most susceptible lines (bottom 10%) for common rust in the DTMA panel.

| Name | Pedigree | Common rust score |
| --- | --- | --- |
| DTMA112 | [Pob.SEW-HG"B"c0F39-1-1-1-1xMBR C5 Bc F22-2-1-4-B-B-B-B-2-2-B-B-B/CML442]-1-1 | 1.26 |
| DTMA93 | CML311/MBR C3 Bc F3-1-1-1-B-B-B-B-B | 1.26 |
| DTMA94 | CML311/MBR C3 Bc F95-2-2-1-B-B-B-B-B | 1.26 |
| DTMA156 | CML-484 | 1.30 |
| DTMA97 | MBR C5 Bc F60-2-1-2-B-B-BxCML 384-B-1-2-B-B-B-B | 1.33 |
| DTMA83 | [(P86 S.F*P.S.P.A.A x P.S.P.A.A. TL91A 44-3-1-18-2P-2-1-1-3-1) x A.I.R.L. TL91A 2(3)-1-4-2-2TL-1-1-B]-3-2-3-1 | 1.36 |
| DTMA148 | CML-322 | 1.43 |
| DTMA150 | CML-371 | 1.43 |
| DTMA36 | ZEWAc1F2-254-2-1-B-1-BBB | 1.43 |
| DTMA11 | CIMCALI8843/S9243-BB-#-B-5-1-BB-4-1-3 | 1.45 |
| DTMA221 | DTPWC9-F109-2-6-1-1-B-B-B | 1.46 |
| DTMA25 | [CML444/CML395//DTPWC8F31-1-1-2-2-BB]-4-2-2-2-1-BB-B | 1.46 |
| DTMA91 | CML311/MBR C3 Bc F12-2-2-2-B-B-BB | 1.46 |
| DTMA133 | POB.501c3 F2 13-8-2-1-BBBB | 1.48 |
| DTMA111 | [[KILIMA ST94A]-30/MSV-03-1-10-B-1-B-B-1xP84c1 F27-4-1-6-B-5-B] F8-3-2-2-1 x G16SeqC1F47-2-1-2-1-BBBB-B-xP84c1 F26-2-2-6-B-3-B]-3-1-B/CML395]-1-1 | 1.50 |
| DTMA3 | [(CML395/CML444)-B-4-1-3-1-B/CML395//DTPWC8F31-1-1-2-2]-5-1-2-2-BB | 1.50 |
| DTMA140 | [P44 c8 FS 158-3-2--4-1-B-B X CML-321]F2-38-1-BB | 1.53 |
| DTMA89 | CML311/MBR C2 Bc F4-1-B-B-BB | 1.53 |
| DTMA98 | MBR/MDR C4 Bc F34-1-B-#-1-1-B-B-B-B-B-B-B | 1.53 |
| DTMA109 | [M37W/ZM607#bF37sr-2-3sr-6-2-X]-8-2-X-1-BB-B-xP84c1 F27-4-3-3-B-1-B] F29-1-1-1-7 x [KILIMA ST94A]-30/MSV-03-2-10-B-1-B-B-xP84c1 F27-4-1-6-B-5-B]-1-3-B/CML312SR]-1-1 | 1.56 |
| DTMA200 | CL-02725=P27(FRRS)C1-248-B-1-B*4 | 1.60 |
| DTMA278 | DTPYC9-F46-3-9-1-1-B-B-B | 1.60 |
| DTMA17 | [CML312/CML445//[TUXPSEQ]C1F2/P49-SR]F2-45-3-2-1-BBB]-1-2-1-1-2-BBB-B | 1.63 |
| DTMA233 | DTPYC9-F46-3-4-1-1-B-B-B | 1.63 |
| DTMA224 | DTPWC9-F67-1-2-1-2-B-B-B | 1.66 |
| DTMA35 | P402c2F2-695-2-BB-2-B*4 | 1.66 |
| DTMA12 | CIMCALI8843/S9243-BB-#-B-5-1-BB-4-3-3 | 1.70 |
| DTMA130 | DTPYC9-F46-3-9-1-1-B-B-B | 1.70 |
| DTMA231 | DTPYC9-F143-5-4-1-2-B-B-B | 1.70 |
| DTMA34 | [CML198/ZSR923S4BULK-2-2-X-X-X-X-1-BB]-3-3-1-1-2-B*7 | 1.70 |
| DTMA165 | S87P69Q(SIYF) 109-1-1-4-B | 3.00 |
| DTMA245 | La Posta Seq C7-F64-2-4-1-1-B-B-B | 3.00 |
| DTMA69 | CLA113 | 3.00 |
| DTMA191 | CLQ-RCYQ28=(CLQ6502*CLQ6601)-B-34-2-2-B*6-B | 3.01 |
| DTMA251 | La Posta Seq C7-F18-3-2-1-1-B-B-B | 3.01 |
| DTMA257 | La Posta Seq C7-F86-3-1-1-1-B-B-B | 3.01 |
| DTMA243 | La Posta Seq C7-F31-2-4-1-1-B-B-B | 3.03 |
| DTMA88 | CML311/MBR C2 Bc F41-2-BBBBB-B-B | 3.05 |
| DTMA57 | CLA18 | 3.06 |
| DTMA183 | DTMA183 | 3.08 |
| DTMA164 | [89[G25Qc1(STE)18S5/Mo176 o2/o2 2-BB]-B-4-4-1-1-4-B*7 | 3.10 |
| DTMA38 | DTMA38 | 3.13 |
| DTMA223 | DTPWC9-F2-3-2-1-1-B-B-B | 3.13 |
| DTMA208 | CL-G1632=G16C20H144#-3-3-1-B*7-B | 3.16 |
| DTMA215 | CML421=P31DMR#1-55-2-3-2-1-B*18-B | 3.19 |
| DTMA74 | CLA157 | 3.19 |
| DTMA170 | CML-326 | 3.23 |
| DTMA153 | CML-330 | 3.24 |
| DTMA212 | CL-G1839=G18SeqC3-17-1-1-2-2-B*5 | 3.26 |
| DTMA56 | CLA12 | 3.33 |
| DTMA269 | DTPWC9-F2-3-2-1-B-B-B | 3.39 |
| DTMA270 | DTPWC9-F32-1-5-1-B-B-B | 3.39 |
| DTMA168 | CML-323 | 3.43 |
| DTMA73 | CLA156 | 3.46 |
| DTMA60 | CLA37 | 3.53 |
| DTMA85 | (200-6 x GUAT189)(51-2-1)F1-B-xP84c1 F26-2-2-4-B-2-B] F102-1-2-2-3 x [KILIMA ST94A]-30/MSV-03-2-10-B-1-B-B-xP84c1 F27-4-1-6-B-5-B]-2-2-B-B-B | 3.58 |
| DTMA63 | CLA46 | 3.98 |
| DTMA62 | CLA44 | 3.99 |
| DTMA72 | CLA155 | 4.06 |
| DTMA71 | CLA154 | 4.13 |
