# Supplementary material for: Genetic Dissection of Quantitative Resistance to Common Rust (Puccinia sorghi) in Tropical Maize (Zea mays L.) by Combined Genome-Wide Association Study, Linkage Mapping, and Genomic Prediction
Source: Front Plant Sci. 2021 Jul 2;12:692205. doi: 10.3389/fpls.2021.692205 (PMC8284423; doi:10.3389/fpls.2021.692205)
Supplement: Supplementary file 4 [file Table_3.DOCX]

Table S3. The most resistant (top 10% ) and most susceptible lines (bottom 10%) for common rust in the bi-parental doubled haploid (DH1) population.

| Name | Pedigree | Common rust score |
| --- | --- | --- |
| GMPGBS71 | (LA POSTA SEQ. C7 F64-2-6-2-2-B-B-B / CML-495 )DH253-B | 1.73 |
| GMPGBS122 | (LA POSTA SEQ. C7 F64-2-6-2-2-B-B-B / CML-495 )DH181-B | 1.87 |
| GMPGBS139 | (LA POSTA SEQ. C7 F64-2-6-2-2-B-B-B / CML-495 )DH210-B | 1.87 |
| GMPGBS41 | (LA POSTA SEQ. C7 F64-2-6-2-2-B-B-B / CML-495 )DH139-B | 1.87 |
| GMPGBS98 | (LA POSTA SEQ. C7 F64-2-6-2-2-B-B-B / CML-495 )DH108-B | 1.87 |
| GMPGBS106 | (LA POSTA SEQ. C7 F64-2-6-2-2-B-B-B / CML-495 )DH112-B | 1.92 |
| GMPGBS150 | (LA POSTA SEQ. C7 F64-2-6-2-2-B-B-B / CML-495 )DH52-B | 1.92 |
| GMPGBS168 | (LA POSTA SEQ. C7 F64-2-6-2-2-B-B-B / CML-495 )DH295-B | 1.92 |
| GMPGBS183 | (LA POSTA SEQ. C7 F64-2-6-2-2-B-B-B / CML-495 )DH243-B | 1.92 |
| GMPGBS192 | (LA POSTA SEQ. C7 F64-2-6-2-2-B-B-B / CML-495 )DH157-B | 1.92 |
| GMPGBS92 | (LA POSTA SEQ. C7 F64-2-6-2-2-B-B-B / CML-495 )DH44-B | 1.93 |
| GMPGBS128 | (LA POSTA SEQ. C7 F64-2-6-2-2-B-B-B / CML-495 )DH131-B | 1.96 |
| GMPGBS15 | (LA POSTA SEQ. C7 F64-2-6-2-2-B-B-B / CML-495 )DH237-B | 1.96 |
| GMPGBS156 | (LA POSTA SEQ. C7 F64-2-6-2-2-B-B-B / CML-495 )DH37-B | 1.96 |
| GMPGBS179 | (LA POSTA SEQ. C7 F64-2-6-2-2-B-B-B / CML-495 )DH102-B | 1.96 |
| GMPGBS44 | (LA POSTA SEQ. C7 F64-2-6-2-2-B-B-B / CML-495 )DH202-B | 1.96 |
| GMPGBS94 | (LA POSTA SEQ. C7 F64-2-6-2-2-B-B-B / CML-495 )DH64-B | 1.96 |
| GMPGBS135 | (LA POSTA SEQ. C7 F64-2-6-2-2-B-B-B / CML-495 )DH9-B | 2.53 |
| GMPGBS52 | (LA POSTA SEQ. C7 F64-2-6-2-2-B-B-B / CML-495 )DH18-B | 2.53 |
| GMPGBS87 | (LA POSTA SEQ. C7 F64-2-6-2-2-B-B-B / CML-495 )DH3-B | 2.53 |
| GMPGBS43 | (LA POSTA SEQ. C7 F64-2-6-2-2-B-B-B / CML-495 )DH142-B | 2.57 |
| GMPGBS1 | (LA POSTA SEQ. C7 F64-2-6-2-2-B-B-B / CML-495 )DH34-B | 2.58 |
| GMPGBS166 | (LA POSTA SEQ. C7 F64-2-6-2-2-B-B-B / CML-495 )DH187-B | 2.58 |
| GMPGBS31 | (LA POSTA SEQ. C7 F64-2-6-2-2-B-B-B / CML-495 )DH137-B | 2.58 |
| GMPGBS195 | (LA POSTA SEQ. C7 F64-2-6-2-2-B-B-B / CML-495 )DH277-B | 2.63 |
| GMPGBS64 | (LA POSTA SEQ. C7 F64-2-6-2-2-B-B-B / CML-495 )DH185-B | 2.63 |
| GMPGBS9 | (LA POSTA SEQ. C7 F64-2-6-2-2-B-B-B / CML-495 )DH122-B | 2.63 |
| GMPGBS196 | (LA POSTA SEQ. C7 F64-2-6-2-2-B-B-B / CML-495 )DH94-B | 2.64 |
| GMPGBS45 | (LA POSTA SEQ. C7 F64-2-6-2-2-B-B-B / CML-495 )DH105-B | 2.68 |
| GMPGBS111 | (LA POSTA SEQ. C7 F64-2-6-2-2-B-B-B / CML-495 )DH11-B | 2.77 |
| GMPGBS127 | (LA POSTA SEQ. C7 F64-2-6-2-2-B-B-B / CML-495 )DH276-B | 2.82 |
| GMPGBS160 | (LA POSTA SEQ. C7 F64-2-6-2-2-B-B-B / CML-495 )DH6-B | 2.82 |
| GMPGBS121 | (LA POSTA SEQ. C7 F64-2-6-2-2-B-B-B / CML-495 )DH296-B | 2.87 |
| GMPGBS155 | (LA POSTA SEQ. C7 F64-2-6-2-2-B-B-B / CML-495 )DH16-B | 2.87 |
| GMPGBS18 | (LA POSTA SEQ. C7 F64-2-6-2-2-B-B-B / CML-495 )DH146-B | 2.87 |
| GMPGBS109 | (LA POSTA SEQ. C7 F64-2-6-2-2-B-B-B / CML-495 )DH67-B | 3.10 |
| GMPGBS69 | (LA POSTA SEQ. C7 F64-2-6-2-2-B-B-B / CML-495 )DH82-B | 3.10 |
