# Supplementary material for: Genetic Dissection of Quantitative Resistance to Common Rust (Puccinia sorghi) in Tropical Maize (Zea mays L.) by Combined Genome-Wide Association Study, Linkage Mapping, and Genomic Prediction
Source: Front Plant Sci. 2021 Jul 2;12:692205. doi: 10.3389/fpls.2021.692205 (PMC8284423; doi:10.3389/fpls.2021.692205)
Supplement: Supplementary file 5 [file Table_4.DOCX]

Table S4. Summary information of GBS-derived SNPs in the Drought Tolerant Maize for Africa (DTMA) panel and the bi-parental doubled haploid (DH1) population before and after filter.

| Population | No. of SNPs | | Missing rate(%) | | Heterozygosity rate(%) | | Average minor allele frequency | |
| --- | --- | --- | --- | --- | --- | --- | --- | --- |
|  | Before filter | After filter | Before filter | After filter | Before filter | After filter | Before filter | After filter |
| DTMA | 955690 | 187409 | 15.79 | 7.33 | 1.49 | 2.83 | 0.09 | 0.18 |
| DH1 | 955690 | 31194 | 42.53 | 9.73 | 0.39 | 3.17 | 0.04 | 0.42 |
